# Morphology Control in a Dual-Cure System for Potential Applications in Additive Manufacturing

**DOI:** 10.3390/polym11030420

**Published:** 2019-03-05

**Authors:** Jonathan A. Campbell, Harrison Inglis, Elson Ng WeiLong, Cheylan McKinley, David A. Lewis

**Affiliations:** 1 Institute for Nanoscale Science and Technology, Flinders University, Sturt Road, Bedford Park SA 5042, Australia; ingl0049@uni.flinders.edu.au (H.I.); cheylan.mckinley@flinders.edu.au (C.M.); david.lewis@flinders.edu.au (D.A.L.); 2 School of Engineering, Nanyang Polytechnic, Singapore 569830, Singapore; Elsonngweilong@hotmail.com

**Keywords:** additive manufacturing, photopolymerisation, dimethacrylate, epoxy, interpenetrating polymer network, IPN, DMTA, morphology

## Abstract

The polymerisation, morphology and mechanical properties of a two-component in-situ reacting system consisting of a rubbery dimethacrylate and a rigid epoxy polymer were investigated. The methacrylate component of the mixture was photocured using UV light exposure and, in a second curing process, the mixture was thermally postcured. The polymers formed a partially miscible system with two glass transition temperature (*T*_g_) peaks measured using dynamic mechanical thermal analysis (DMTA). The composition and relative rate of reaction of the two orthogonal polymerisations influenced the extent of miscibility of the two polymer-rich phases and the samples were transparent, indicating that the two phases were finely dispersed. The addition of a glycidyl methacrylate compatibiliser further increased the miscibility of the two polymers. The utility of this polymer system for additive manufacturing was investigated and simulated through layer-by-layer processing of the mixture in two steps. Firstly, the methacrylate component was photocured to solidify the material into its final shape, whilst the second step of thermal curing was used to polymerise the epoxy component. With the use of a simulated photomask, a simple shape was formed using the two orthogonal polymerisation stages to produce a solid object. The mechanical properties of this two-phase system were superior to a control sample made only of the methacrylate component, indicating that some reinforcing due to polymerisation of the epoxy across the interfaces had occurred in the postcuring stage.

## 1. Introduction

The combination of two polymers to impart unique properties is well established. Two polymers can be mechanically blended to form a mixture, however due to the incompatibility of most polymers the properties are often inferior to the components. Compatibilising additives can be used to increase miscibility with a more robust approach involving the chemical linking of the two components, such as in copolymerisation or grafting [1,2,3]. The polymerisation of one or both components in-situ can be used to form an interpenetrating polymer network (IPN) [4]. IPNs provide some novel possibilities for tailoring characteristics of the final polymer system as the sequence of polymerisation and compatibility of the two components can influence the morphology and properties [5].

This ability to tailor properties of IPNs, particularly the dual polymerisation processes that occur in full IPNs, leads to their potential utility in additive manufacturing (AM) processes. AM is attractive for reducing inventory costs, enabling mass customisation and, in particular, making parts that are complex and have shapes that cannot be made by traditional moulding/casting and subtractive manufacturing techniques [6,7]. Beyond the novelty applications that make up a large proportion of the use of AM, most industrial uses are for prototyping and small runs of parts to avoid the costly making of moulds for example (and in some cases the making of moulds). In general, these are mostly for visual rather than structural applications because the mechanical properties are anisotropic [8], so polymer parts made by AM often have inferior mechanical properties than parts made by traditional methods such as injection moulding.

**Figure 1:**
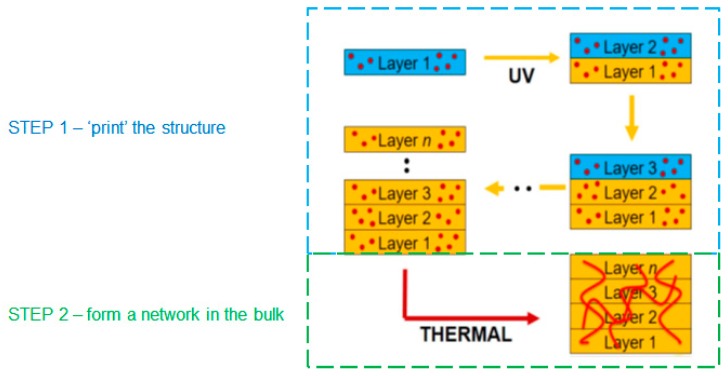
Schematic of the two-stage cure process.

The degree of property anisotropy can vary with the AM technique used, however the properties of the final product are heavily influenced by the large number of interfaces in the structure due to the layer-by-layer deposition process that is the basis of AM techniques [8,9,10]. This typically results in lower strength in the build direction due to weaknesses associated with the multiple interfaces. Materials that cure in two stages have the potential to chemically react across the interfaces formed in each printing step, by utilizing one chemistry to rapidly define the shape of the object and the second chemistry to provide the monolithic properties. This dual-cure approach therefore, provides an opportunity to reduce the significance of interfaces between print layers and to create a structure with greater homogeneity in all directions, as shown in Figure 1.

Various stereo lithographic printing (SLA) techniques, such as digital layer projection (DLP), utilise photoactive liquids and have the advantage of high resolution (down to 50 microns), however the range of available materials is limited [11]. The formation of an IPN structure, in which the two polymer networks are physically intermingled has the advantages of control over phase separation, combination of properties (synergy) and in-situ processability [12] and is therefore, ideally suited to AM processing. Dual-curing systems for additive manufacturing have been investigated by a number of authors. Some examples include an epoxy/methacrylate IPN for use as a matrix in carbon fibre composites made by UV-assisted 3D printing [13,14], silicone-epoxy/acrylate [15], adipic acid glycidyl methacrylate/poly isocyanate [16] and acrylate/epoxy systems with tailored properties through changes in processing conditions [17]. Dual-curing mixtures of acrylates and epoxy materials are common in commercial SLA printing [6], and a dual-cure process was recently commercialised by Carbon Co. [18,19] in their continuous liquid interface process. Additionally, multi-component/dual-curing polymer IPNs have been investigated for a range of applications including acrylate urethane for automotive coatings [20] and have been utilised for the control of reaction exotherms in industrial processing of thermoset resins [21].

This paper reports a proof of concept for this approach with a candidate pair of chemistries that cure orthogonally and have well-separated glass transition temperatures, one being rubbery and the other rigid at ambient temperatures. The monomers were chosen to demonstrate this approach: 9G is a glycol di-methacrylate with an average of 9 glycol units, resulting in a gel point at low conversion which can provide rapid “setting” of the liquid to maintain the accuracy of deposition and can be photocured using a range of photoinitiators. Additionally, 9G has a relatively low viscosity and the glycol units result in compatibility with a wide range of chemistries, making it an ideal candidate for a range of IPNs. Similar chemistry is also available with different numbers of glycol units which provide a pathway to tuning the glass transition temperature (*T*_g_), in this case 9G having a *T*_g_ of 15 °C [22]. The epoxy chosen was bisphenol A diglycidyl ether (DGEBA) cured with 2,2,4-trimethylhexane-1,6-diamine. This material has a *T*_g_ of approximately 65 °C, which provides sufficient separation from the *T*_g_ of 9G to allow determination of the morphology that is formed upon curing by measurement of the two discreet *T*_g_ values for each of these components. The formation of an IPN in this system would be expected to result in superior properties to a phase-separated blend [4,23], potentially combining the attributes of both component polymers and resulting in a material with both high strength due to the epoxy network and toughness due to the rubbery 9G network. Polymer IPN structures containing methacrylate have been investigated for use in dental materials for example [24]. In this case, a full IPN would be formed as both phases are crosslinked thermosets and are mixed in one step [25].

## 2. Materials and Methods

In this study, poly (ethylene glycol dimethacrylate) (9G) was sourced from Shin-Nakamura Chemical (Wakayama, Japan). The thermally-activated epoxy system used was bisphenol A diglycidyl ether and an aliphatic diamine (2,2,4-trimethylhexane-1,6-diamine) grade 301, sourced from Epo-Tek (Billerica, MA, USA). The benzophenone (BP), glycidyl methacrylate (GMA), azobisisobutyronitrile (AIBN) and triethylamine (TEA) were from Sigma Aldrich (Sydney, Australia).

The epoxy methacrylate mixtures were cured under a UV lamp (1 mW/cm^2^, λ_max_ 370 nm) for up to 20 min in the first stage of the curing process. In some circumstances, it was found that the monomers did not fully cure at the sample’s surface, leaving a very thin liquid layer. This was attributed to oxygen inhibition of the methacrylate polymerisation. Previous studies have shown that even a 1% presence of oxygen has a significant impact on the polymerisation by hydroperoxide formation [26]. In order to reduce this effect, the resin was degassed and the photopolymerisation reaction was conducted under inert atmosphere (N_2_) during sample preparation. Triethylamine could also be used to prevent oxygen inhibition through chain transfer, and was used in the photomask experiment. The second stage of the curing process was completed in an oven at a temperature between 65 and 120 °C for 2 h. 

Fourier-transform infrared spectroscopy (FTIR) studies utilised a Nicolet 8700 spectrometer with a diamond ATR stage, scanned at 500–4000 cm^−1^ at a resolution of 2 cm^−1^ using an MCT detector. Dynamic mechanical thermal analysis (DMTA) characterisation was undertaken using a TA Instruments Q800 DMA with a tension clamp, at a heating rate of 3 °C/min. 

Samples for mechanical testing were cast into a Teflon mould with the dimensions defined for tensile specimen ISO 527-2 specimen type 1BA, with a gauge length of 30 mm, a width of 5 mm and a thickness of 2 mm. For a solid tensile sample, the solution was cast into the mould and cured in a single UV exposure and then thermally postcured. For tensile bars with 10 interfaces along the gauge length, each 3 mm-long section was poured and then UV cured individually to simulate a layer-by-layer polymerisation process (using a plug to limit the size of each poured section). Then the whole bar was thermally postcured and the dogbone samples tested in tension using an Instron 4301 at a crosshead speed of 50 mm/min. The results presented are an average of 3 samples.

## 3. Results and Discussion

**Figure 2:**
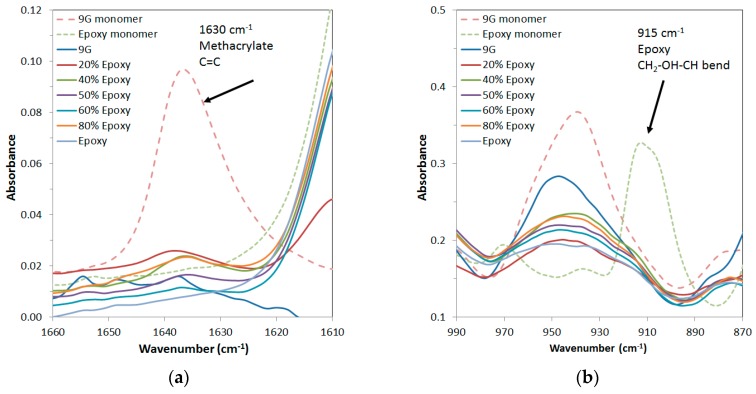
FTIR spectra of epoxy/methacrylate samples: (**a**) Epoxy CH_2_–OH–CH peak and (**b**) methacrylate C=C peak after completion of polymerisation steps.

Mixtures with a range of 9G/epoxy ratios formed a homogeneous solution when mixed and were polymerised in two steps. Initial photocuring of the 9G was followed by thermal postcuring of the sample to polymerise the epoxy component. FTIR analysis of a range of compositions was undertaken to monitor the polymerisation as both the methacrylate and epoxy groups have characteristic absorbances at 1630 cm^−1^ (C=C bond of methacrylate) and 915 cm^−1^ (CH_2_–OH–CH bend of the epoxy group), respectively. Figure 2 shows these bands for the uncured resin starting materials and a range of compositions that were photopolymerised and then subjected to a low temperature curing step at 65 °C. It is clear that these peaks were significantly decreased for all systems, but the methacrylate was not fully cured under these conditions. This is explained by the overlap of the lamp emittance spectra being limited to the high wavelength shoulder of the absorbance spectra of benzophenone, resulting in a slow curing process (for further details see Supplementary Information Figure S1). After postcuring at 120 °C to overcome the limitations of vitrification on conversion, the FTIR peaks for uncured methacrylate were not present in the spectra, indicating that both components were fully polymerised. The DMTA properties did not change after postcuring at 120 °C, indicating that no further polymerisation occurred after this step as a result of further heating.

To extend the shelf life of the monomer mixture and ensure the correct stoichiometry for the epoxy curing step (in order to achieve the expected properties), it is important that the two components polymerise in separate steps and that there should be no reaction between the two systems (orthogonal systems). FTIR indicated that the cure of the methacrylate and the amine are orthogonal and that the Michael addition does not occur. This result was confirmed by 1H NMR of a mixture of these components after 24 h at ambient conditions, which showed that the occurrence of the Michael addition [27] was insignificant (see Supplementary Information Figure S2).

**Figure 3:**
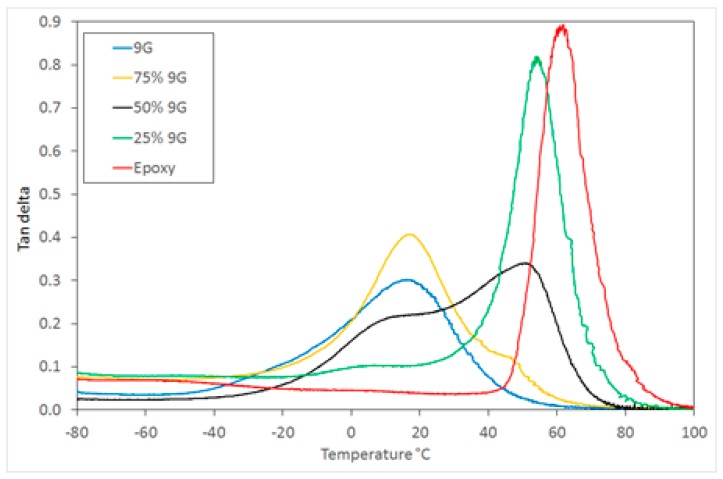
Dynamic mechanical thermal analysis (DMTA) tan δ of the polymerised samples.

DMTA analysis is particularly useful for characterising the glass transition process in multiphase systems and can be used to interpret the phase mixing behaviour and resulting microstructure. Figure 3 shows the tan δ response for a range of postcured compositions, including pure 9G and epoxy, which have *T*_g_ values at 15 and 62 °C respectively. The development of two glass transitions was achieved after the second (thermal) curing step when the epoxy monomer is polymerised (see Supplementary Information Figure S3).

The DMTA analysis showed that the *T*_g_ for the two phases are intermediate between those of the component polymers, indicating that there is some miscibility between the two components, but sufficient phase separation to result in two glass transitions. These are attributed to two component-rich phases with a broad composition range (see Figure 3). The samples produced were visually transparent with only a small amount of haze visible, indicating that a finely dispersed network of two polymer-rich phases has been produced [28] (transparency would be achieved at less than 100 nm, or approximately one quarter the wavelength of light). 

**Figure 4:**
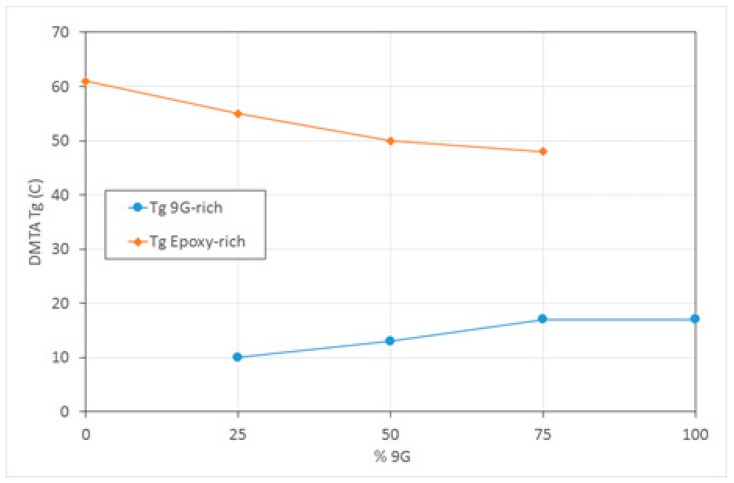
DMTA *T*_g_ (tan δ peak temperature) of epoxy-rich and 9G-rich phases.

The *T*_g_ (tan δ peak) values of the epoxy-rich phase are slightly lower than the pure epoxy phase and decrease further as the amount of methacrylate increases, indicating that there is some miscibility of the 9G in the epoxy (Figure 4). The *T*_g_ of the 9G-rich phase reduces slightly with the increasing amount of epoxy present in the mixture, which could be due to the diffusion limitation of the polymerisation process, resulting in a lower molecular weight polymer. It has been found that the presence of a second polymerising species can have a significant impact on the polymerisation kinetics and conversion, including during the formation of epoxy-acrylate IPNs [29].

**Figure 5:**
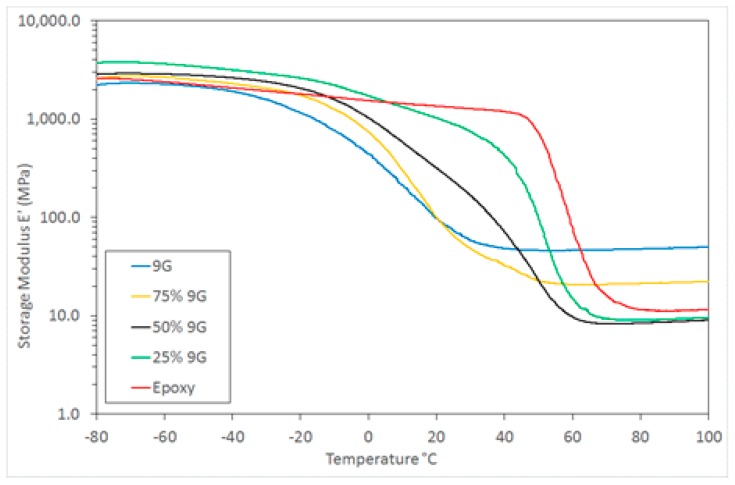
DMTA storage modulus for postcured samples.

**Figure 6:**
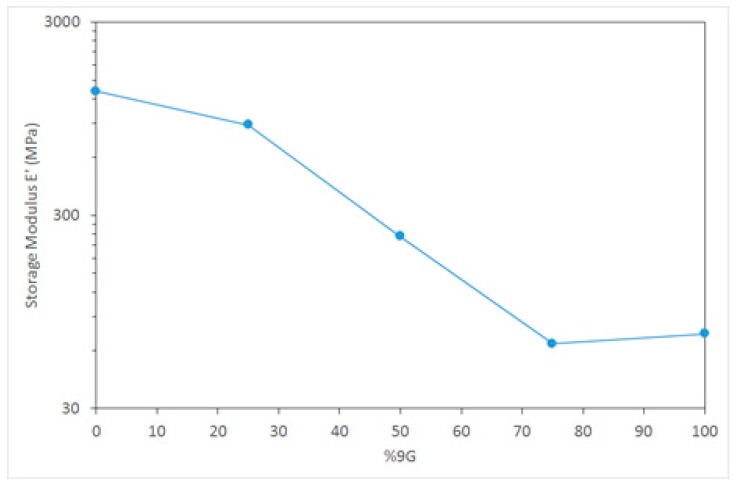
DMTA storage modulus for postcured samples at 25 °C.

Despite the combination of these general classes of polymers being investigated previously, research has generally been concerned with a combination of two rigid, high *T*_g_ polymers [30]. In this case, by using a combination of high and low *T*_g_ polymers the opportunity exists to not only tailor the morphology of these two distinct systems, but to manipulate the mechanical properties over a wide range. This is illustrated by the range of DMTA storage modulus behaviour for these samples (Figure 5) and particularly the wide range of modulus values available at an ambient temperature (25 °C in this example) as shown in Figure 6.

The timing of the two components’ polymerisation is important in the formation of the microstructure in the network. For example, in a study on dimethacrylate/epoxy IPNs [31] the formation of a single phase was achieved by curing the epoxy first, but a two-phase morphology was formed when photo-curing the methacrylate component first. This was attributed to the epoxy forming a gel at a high conversion (57% theoretical value [32]), which allows the epoxy to diffuse out of the mixture during polymerisation. Conversely, the dimethacrylate gelled at only 1% conversion [33], locking the epoxy within the network and forming a single phase. A similar effect was reported for a polyurethane/polyacrylate IPN formation, where it was shown that simultaneous polymerisation of an IPN resulted in less phase separation than sequential polymerisation [34]. This was explained by the faster cure causing a quicker interlocking of polymer networks in relation to the phase separation process, but a slower sequential polymerisation allowed one phase to remain more mobile while the first phase polymerised, leading to greater phase separation. The rate at which these individual processes take place is therefore expected to influence the microstructure and properties of the final network [29]. 

**Figure 7:**
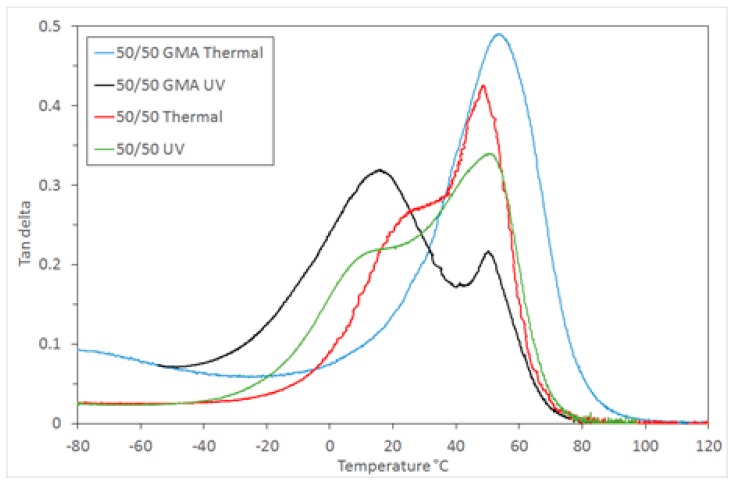
DMTA tan δ for 50/50 compositions initiated either thermally or by UV initiator, and with/without compatibiliser.

The effect of the two components’ relative rate of curing on the morphology in the 9G/epoxy system was also studied here. Samples were prepared using a thermal curing process for both components by introducing a thermal initiator (AIBN) to replace the photoinitiator used for methacrylate polymerisation. This provided an opportunity to investigate how the microstructure develops when the timing of both polymerisations is similar. DMTA analysis of the photocured and thermally cured 50/50 mixtures showed that both contain two phases, however the *T*_g_ values in the thermally cured sample are closer together, indicating a greater degree of miscibility of each polymer in the other (Figure 7). This is a result of the faster curing process, limiting the time available for phase separation. 

The addition of compatibiliser can influence the miscibility of the two components and the resulting microstructure [2]. To assess the effect of a compatibilising component in this system glycidyl methacrylate was chosen, as it has both a methacrylate and an epoxy moiety, and so can potentially participate in the polymerisation of both the components of this system. This also has the potential to widen the process window for such a polymerising system by reducing the effect of time between the two stages of curing. The DMTA analysis of the 50/50 sample with 5 wt % GMA added shows that a miscible single phase is formed when the polymerisation is simultaneous (thermal initiation) and a partially-miscible two phase structure is formed when photocuring is used for the first step (Figure 7). The level of miscibility for the photocured system is also different to the same mixture without GMA. The incorporation of GMA leads to a larger 9G-rich phase *T*_g_ peak and a smaller epoxy-rich phase peak, indicating that whilst there are still two phases, the 9G phase dominates the thermomechanical response, possibly due to the GMA being more readily incorporated into the 9G-rich phase when photocuring occurs first. The shift in *T*_g_ of the two component-rich phases is similar in both cases, but the breadth of the tan δ peak is greater for the GMA system, indicating a wider range of miscible states. The samples produced were highly transparent, indicating the increased compatibility of the two networks.

**Figure 8:**
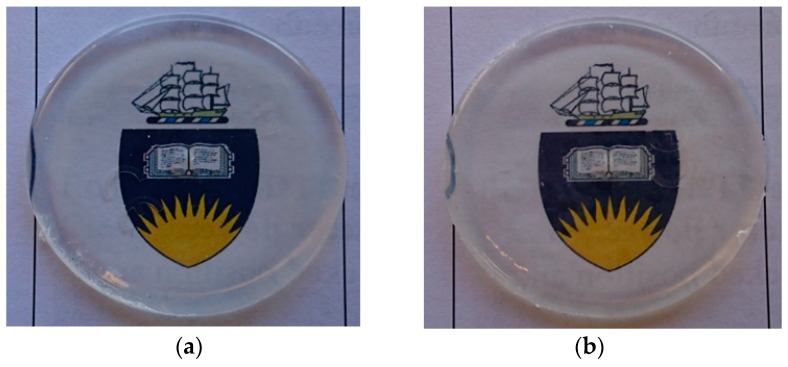
50/50 + 5 wt % glycidyl methacrylate (GMA) composition after two-step polymerisation: (**a**) 1 layer and (**b**) 3 layers.

The GMA compatibilised polymer system was chosen for further practical simulations of layer-by-layer printing. Samples were prepared with 1 and 3 layers of the 50/50 + 5 wt % GMA composition to illustrate that the process of adding more layers does not change the way in which the polymerisations progress. In both cases, the samples were transparent and similar in physical appearance (Figure 8).

**Figure 9:**
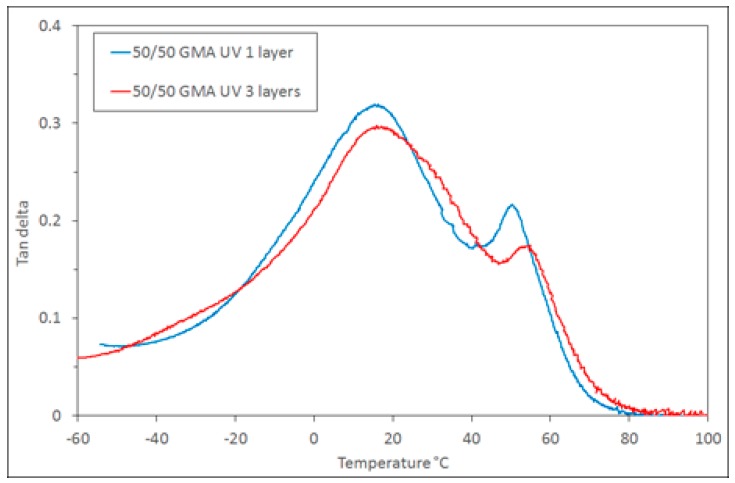
DMTA tan δ of the 1 layer and 3 layer 50/50 GMA samples.

The DMTA analysis of these samples also shows that the resulting polymer is similar, with the broad 9G-rich phase tan δ peak *T*_g_ at 15 °C and the epoxy-rich phase *T*_g_ at 50–55 °C (Figure 9). The tan δ curves show that the phase behaviour (which implies the curing behaviour) remains similar as more layers are added. This illustrates the applicability of this polymerising system for use in the types of layer-by-layer techniques employed in additive manufacturing.

**Figure 10:**
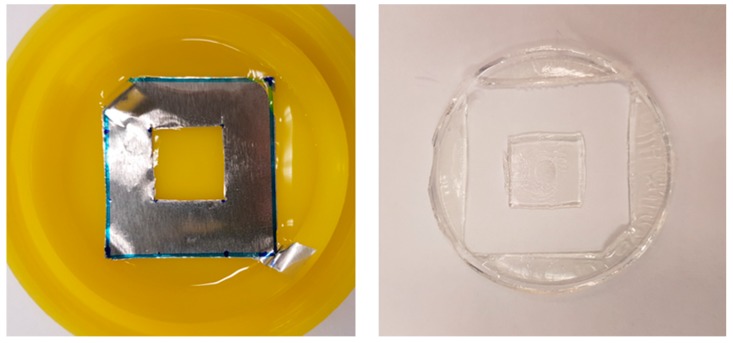
Simple photomask and the resulting part made by two-step polymerisation (50/50 + 5% GMA).

In order to illustrate the ability of this two-step curing system to retain its shape after the first photocuring stage, the 50/50 9G/epoxy + 5 wt % GMA composition was formed into a solid shape by using a simple square shaped photomask (Figure 10). Three layers of monomer mixture were manually added and photocured after each layer, and the part was then thermally cured as a whole. This simple experiment shows that the shape was maintained when the system underwent only the photocuring step, and that subsequent layers can be added on top. Postcuring at elevated temperature then ensured polymerisation of the epoxy phase within the network formed by the methacrylate phase.

Mechanical properties of the polymerised polymer system were investigated by tensile testing dogbone samples made by casting the mixture into a mould. The failure stress, maximum strain and modulus for 100% 9G and 50% 9G samples made as both a solid bar and as a bar containing 10 interfaces were evaluated. The comparison of the absolute results shows that all three parameters were lower for the sample with interfaces (Table 1). This is expected because the tensile test is very defect sensitive, so the presence of many interfaces would be expected to reduce the absolute values of these parameters. However, if the difference between the solid and interface samples are compared as a ratio (the value for the 50% 9G as a proportion of the value for the 100% 9G sample), the 50% 9G composition has higher relative values in the samples containing interfaces. 

For example, the failure stress of the solid sample made with 50% 9G is 20% of the value of the 100% 9G solid sample. For the samples made with interfaces, the 50% 9G failure strength is 32% of that of the 100% 9G sample. Therefore, by removing the effect of the interfaces (by using a control made in the same way in each case), the 50% 9G composition has a comparatively higher failure strength than the 100% 9G composition. 

Similarly, the elongation at break and the tensile modulus values are comparatively higher for the 50% 9G composition in the interface samples. However, within error (using the standard deviation) only the tensile strength and elongation at break values are sufficiently separated to illustrate this trend. It is not generally expected that the elongation and failure strength both increase when the composition of a blend is changed, but can be observed when an IPN is formed [35].

This shows that the epoxy phase, although cured in the second stage of the polymerisation, contributes to the strength throughout the sample, including across the interfaces. This is evident in that a 3-dimensional network of both the polymers has formed, which extends across the interfaces within the sample after thermal treatment in the second curing stage.

## 4. Conclusions

The dimethacrylate/epoxy system was polymerised using a dual-cure process where the methacrylate component of the mixture was photocured using UV light exposure and in a second curing process the mixture was thermally postcured. The polymers formed a partially miscible system with two *T*_g_ peaks measured using DMTA. The composition and relative rate of reaction of the two orthogonal polymerisations influenced the extent of miscibility of the two polymer-rich phases, and indicated a broad range of miscibility. A wide range of storage modulus values were obtained at 25 °C for various compositions, illustrating the ability to control the properties by the ratio of the two components. The addition of a glycidyl methacrylate compatibiliser further influenced the miscibility of the two polymers. Samples were transparent, indicating that the two phases were finely dispersed. 

The utility of this polymer system for additive manufacturing was investigated by simulated layer-by-layer processing of the mixture in two steps. Photocuring of the methacrylate component was first used to solidify the material into its final shape, whilst the second step of thermal curing was used to polymerise the epoxy component. Use of a simple photomask simulation showed proof of concept for the layer-by-layer application of this polymer system, with a simple shape able to be formed using the two orthogonal polymerisation stages to produce a solid object. The mechanical properties of tensile bars incorporating 10 interfaces were investigated as a model for an additive manufactured part. The tensile strength and elongation at the break of the 50/50 composition were superior to a control sample made only of the methacrylate component, indicating that some reinforcing polymerisation across the interfaces had occurred in the postcuring stage.

## Figures and Tables

**Table 1:** Tensile properties of 9G and 9G/epoxy as a solid bar or 10-layer bar.

Composition	Tensile Strength (MPa)		Elongation at Break (%)		Tensile Modulus (MPa)	
	Solid	10 Interfaces	Solid	10 Interfaces	Solid	10 Interfaces
100%9G	5.4	2.8	17.3	11.3	68	54
*stdev*	*1.3*	*0.6*	*2.7*	*2.0*	*12*	*12*
50%9G	1.1	0.9	19.0	13.7	13	15
*stdev*	*0.11*	*0.06*	*2.3*	*3.0*	*1.4*	*2.3*
ratio	20%	32%	110%	121%	19%	28%

## Supplementary Materials

The following are available online at https://www.mdpi.com/2073-4360/11/3/420/s1, Figure S1: Absorbance spectra for 9G, 9G + 1% BP, 9G + 1% BP diluted, and emittance spectra for UV lamp, Figure S2: 1H NMR spectra of 9G monomer and diamine after 24 h, Figure S3: A 50/50 mixture of 9G and Epoxy before and after postcuring at 120 °C.
