# Supplementary material for: Morphology Control in a Dual-Cure System for Potential Applications in Additive Manufacturing
Source: Polymers (Basel). 2019 Mar 5;11(3):420. doi: 10.3390/polym11030420 (PMC6473805; doi:10.3390/polym11030420)
Supplement: Supplementary file 1 [file polymers-11-00420-s001.pdf]

Supplementary Information 1

The peak emittance wavelength of the UV lamp and the peak absorbance of the benzophenone photoinitiator in 9G are shown in Figure S1. The 100x diluted mixture was also measured to show the overall shape of the curve. The overlap of the lamp emittance spectra is limited to the high wavelength shoulder of the absorbance spectra of benzophenone up to 395nm. This resulted in a slower curing process than if the two wavelength maxima had been aligned closely.

The UV-vis spectra were analysed using a Varian Cary 50 UV-vis spectrophotometer with 1 cm pathlength cuvette.

Characterization of the spectral output of the light source was undertaken using an Ocean Optics Jaz Spectrometer, equipped with a JAZ-PX module a pulsed xenon lamp (200 – 1100 nm operating range) operating in reflectance mode with a 10 msec integration time.

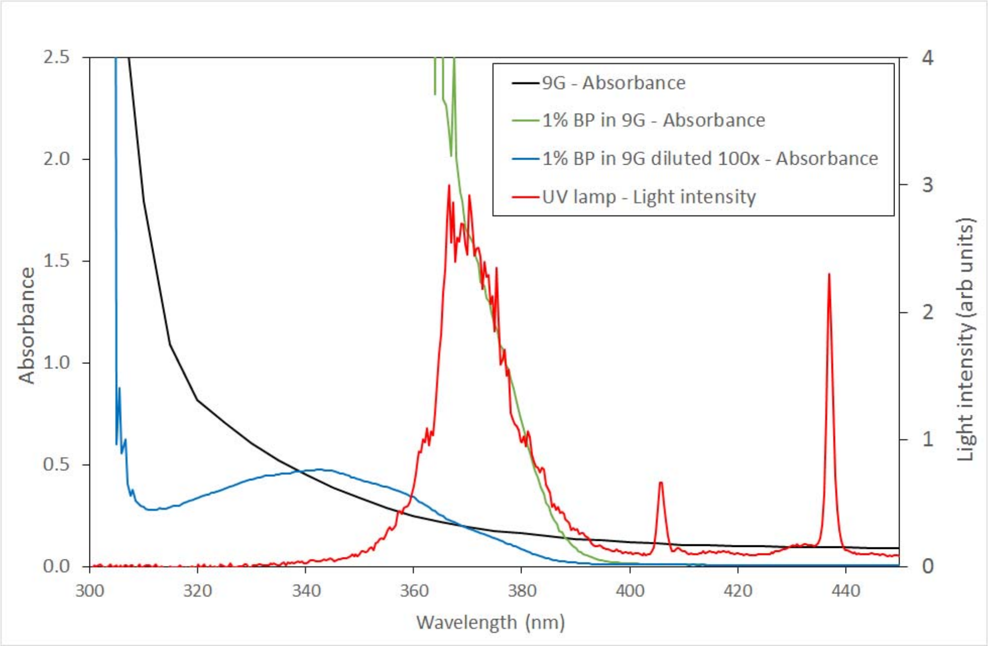

Figure S1. Absorbance spectra for 9G, 9G + 1% BP, 9G + 1%BP diluted, and emittance spectra for UV lamp.

Supplementary Information 2

The Michael reaction is a 1, 4 conjugate addition of an amine group with an alkene. In this system it is possible that there is reaction between the amine and 9G polymer, reducing the orthogonality of the two reaction mechanisms. Escalante *et al.* [27] discuss the reaction conditions required for high yields that include 5 days, 65°C oil bath, 73% yield, or Microwave irradiation in methanol, 83-98% yield.

In this project the mixtures were held at room temperature for no longer than an hour before initial UV curing, providing much less opportunity for this reaction to occur. In order to test the extent of possible Michael reaction in this system, a mixture of 3g of 9G monomer and 0.6 g of diamine were held at ambient temperature for 24 hours (longer reaction time than used for sample prep) and then analysed using 1H NMR.

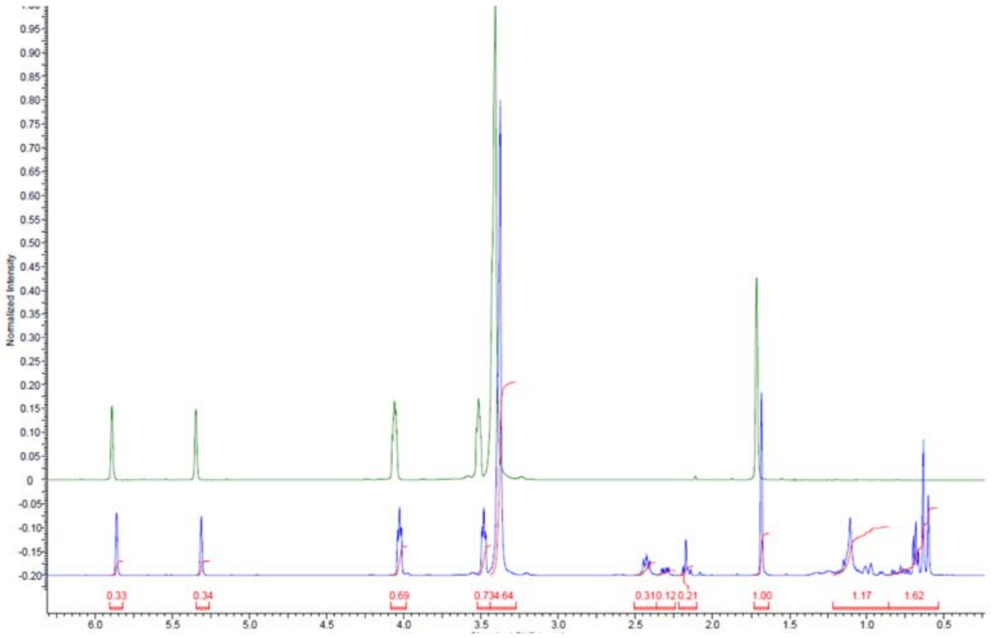

Figure S2. 1H NMR spectra of 9G monomer and diamine after 24 hours.

Three hydrogen regions are identifiable from the structure and can be associated the peaks as thus:

Red: $-CH_3$, δ ≈1.7ppm. Blue: $-CH_2$, δ≈5.3/5.85ppm. Green: $-O-CH_2-CH_2-$, δ≈3.4/4.0 ppm.

These attributions are consistent with 1H NMR theory and literature values for chemical shift of 9G. There should be a reduction in the intensity of the peaks contributed by the $-CH_2$ peaks. The ratio of the $-CH_3$ and $-CH_2$ peaks were taken from both the pure sample and the mixture and compared. A change in 0.01 intensity of the ratio of the key peaks was examined. This experiment was an exaggeration of the reaction conditions of the project. The alteration to the signal relating to the significant $-CH_2$ contribution was deemed insignificant. The Michael addition was considered to be non-applicable under the conditions of the project.

Supplementary Information 3

After the initial photocuring step of the 50/50 w/w mixture, there is a single Tg peak at -20°C which is lower than that expected of 9G alone at 15°C. This is because at this stage only the 9G network has formed, and the epoxy remains mostly uncured as shown by FTIR. The low temperature of the Tg peak is due to the plasticisation of the 9G with uncured epoxy, and the vitrification of the 9G preventing further curing. After heating to 120°C there are two Tg peaks observed indicating that both components have polymerised, and that there are two phases, each rich in one of the component polymers.

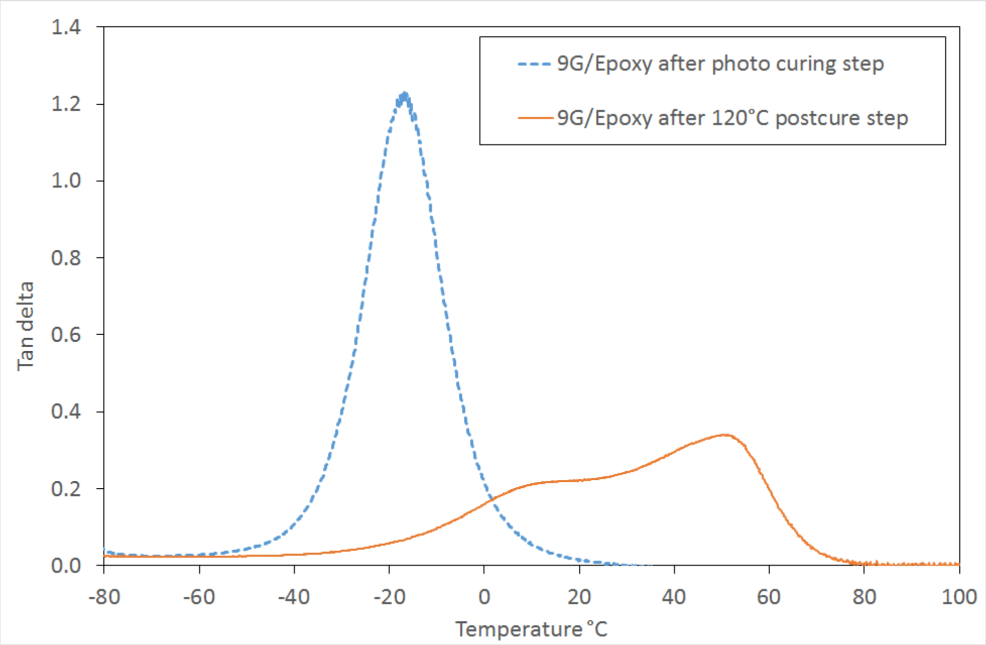

Figure S3. 50/50 mixture of 9G and epoxy before and after post-curing at 120°C.
